# Sow vaccination against virulent *Glaesserella parasuis* shapes the nasal microbiota of their offspring

**DOI:** 10.1038/s41598-022-07382-2

**Published:** 2022-03-01

**Authors:** Miguel Blanco-Fuertes, Florencia Correa-Fiz, Sergi López-Serrano, Marina Sibila, Virginia Aragon

**Affiliations:** 1 IRTA, Centre de Recerca en Sanitat Animal (CReSA, IRTA-UAB), Campus de la Universitat Autònoma de Barcelona, 08193 Bellaterra, Spain; 2 OIE Collaborating Centre for the Research and Control of Emerging and Re-Emerging Swine Diseases in Europe (IRTA-CReSA), Bellaterra, Barcelona, Spain

**Keywords:** Metagenomics, Vaccines, Pathogens

## Abstract

*Glaesserella parasuis* is the etiological agent of Glässer’s disease, a common pathology in the pork industry with higher prevalence in the postweaning period. Vaccination is one of the strategies to control this disease. Here, we investigated the effect that sow vaccination against virulent strains of *G. parasuis* had in the nasal microbiota of their offspring. Nasal swabs from fifteen days-old piglets from vaccinated (vs-P, n = 11) and unvaccinated sows (cs-P, n = 11) were obtained and DNA was extracted for 16S amplicon sequencing. Microbiota composition was different, with lower diversity in vs-P, and a strong clustering of the groups in beta diversity analysis. Among the 1509 sequences associated to either study group, all the sequences classified as *G. parasuis* (10 ASVs) had lower relative abundance in the vs-P group. A list of 32 inferred metabolic pathways were statistically different between groups. A distinctive structure of the two microbial networks was detected, with modules in the cs-P not conserved in the vs-P network. In conclusion, vaccination of the sows had a large effect in the microbiota composition of their offspring that went beyond the effect on the targeted pathogen. The mechanisms underneath these changes may include alteration of the microbiota network due to the elimination of the targeted pathogen and/or immunological changes.

## Introduction

*Glaesserella parasuis* is the etiological agent of Glässer’s disease, an infectious disease with an important economic impact on swine production^1^. This microorganism is an early colonizer of the upper respiratory tract and is part of the healthy nasal microbiota during all the stages of pig’s life. In order to reduce the prevalence of Glässer’s disease, different strategies have been used. Antimicrobial treatments and vaccines are the main tools to control disease^2^, although the global concern about rising antimicrobial resistances makes vaccination the method of choice. The natural source of this microorganism for piglets resides in the dams and colonization is known to occur very early in life^3^. Vaccination of the sows increases the specific antibodies transferred to their offspring through colostrum intake^4^ and may delay the colonization by *G. parasuis*^3^. Thus, sow vaccination can be a good strategy to decrease the prevalence of the virulent strains of this microorganism in the offspring, when using a vaccine that specifically targets these strains^5^.

The microbiota, known as the set of microorganisms that inhabit a host^6,7^, has different essential functions for the host such as nutrition, preservation of the mucosal homeostasis, prevention of pathogen colonization, and maturation of the immune system^8^. Nasal microbiota, as gut microbiota, is affected by a wide range of factors in piglets, such as age, contact with the mother or environment^9–11^. Several studies have examined the impact of the pig microbiome on the immune response to vaccination^12,13^, but the impact of vaccination on the microbiota had not been extensively analyzed, with a few studies focusing on gut microbiota^14–16^. No information is yet available about the effect that specific stimulation of the immune system by vaccines could have on the nasal microbiota of the piglet.

Here, we report the effect of vaccination of sows against virulent *G. parasuis* on the nasal microbiota composition of their offspring. A reduction in the relative abundance of *G. parasuis* sequences was found in the samples from the piglets derived from vaccinated sows. In addition, other differences in the microbial composition were detected; especially, on the most relatively abundant bacterial taxa, as well as in inferred pathways, which were divergent between piglets born to vaccinated or non-vaccinated sows. Correlation Network Analysis (CNA) resulted in two complex networks with a different number of nodes and edges. The structure of the modules containing the most connected nodes in the co-occurrent ASVs control network was not conserved in the network from vaccinated sows.

## Results

### Sequencing throughput stats

After the denoising and quality control steps, a total of 9817 features were found in the 22 samples of the study. A total of 9,202,583 sequences were analyzed with a minimum ASVs frequency per sample of 211,733 and maximum of 649,315, being the mean 418,299. The ASVs had a mean length of 414 bp (range 236–444), where 98% of the sequences were 428 bp length. After processing, 73% of the raw sequences passed the quality-control steps and were used in the downstream analyses.

### Sow vaccination decreased diversity in the nasal microbiota of the offspring

**Figure 1:**
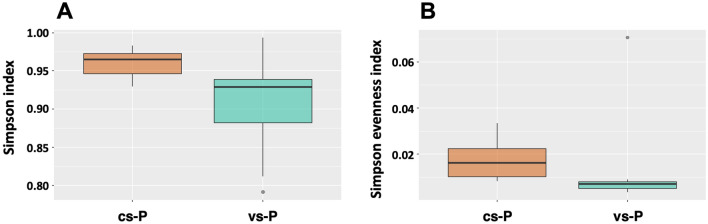
Alpha diversity measured as Simpson index and calculated as 1-D (**A**) and Simpson evenness index (**B**). Samples from nasal microbiota of 15-day-old piglets born to control unvaccinated sows (cs-P, orange boxes) and to vaccinated sows (vs-P, green boxes) with a vaccine against virulent *G. parasuis* were analyzed. These two indexes resulted statistically significant (*P* < 0.05).

Richness and alpha diversity metrics (Observed Features, Simpson, Shannon, Faith phylogenetic diversity and Chao indexes) were calculated and compared between piglets born to vaccinated sows (vs-P) and to non-vaccinated sows (cs-P). Nasal microbiota from vs-P was significantly less diverse than cs-P when using Simpson diversity index and Simpson evenness index (Fig. 1). The rest of indexes showed the same tendency although they did not detect statistically significant differences between both groups.

**Figure 2:**
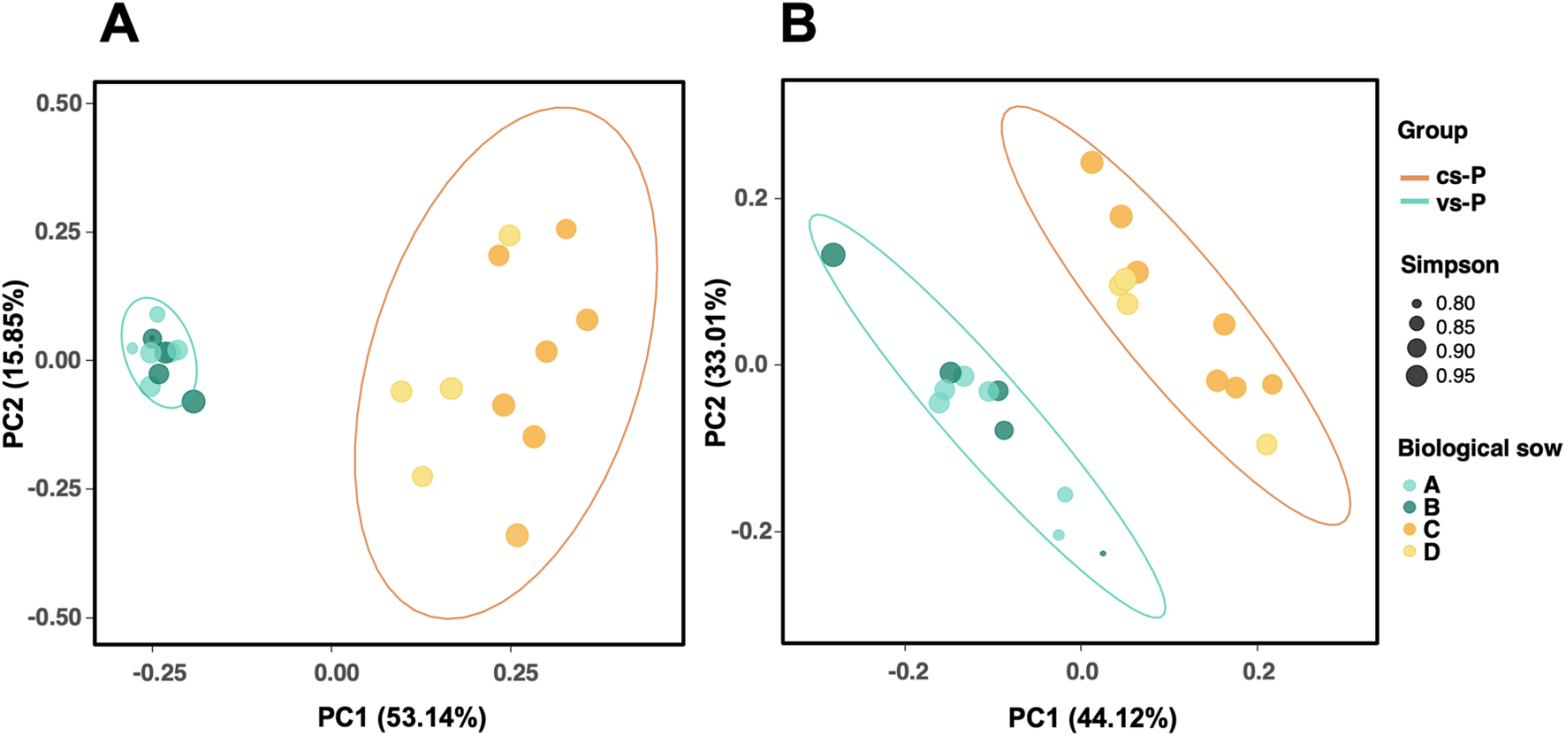
PCoA plots representing the beta diversity of the nasal microbiota of piglets from vaccinated (green) and control unvaccinated sows (orange). Bray Curtis distance matrix is represented in the left (**A**) and weighted Unifrac index on the right (**B**). Each sample is colored based on the biological sow, and the size of the symbol is proportional to the Simpson index (alpha diversity).

The composition of the nasal microbiota evaluated through several beta diversity metrics showed a divergency between the vs-P and cs-P groups and this divergency was statistically significant when evaluated either quantitatively or qualitatively (*P* < 0.001, PERMANOVA test). The first axis in the PCoA (PC1) using Bray Curtis dissimilarity index, captured the highest amount of variation of the input data (53.14%; Fig. 2A) and a high percentage of the differences were explained by the vaccination of the sows (R^2^ = 51.47%; Adonis function). Spatial representation of the Jaccard distance (a metric that only calculates the presence or absence of the different ASVs in each sample) showed a 16.08% of variation among the samples in the PC1 (Supplementary Fig. S1), which was mainly explained by sow vaccination (R^2^ = 15%, Adonis function). Similar results were also obtained with metrics that include phylogenetic relationships between the microorganisms. Thus, weighted (Fig. 2B) and unweighted (Supplementary Fig. S1) Unifrac showed PC1 with percentages of 44.12%, and 15.52%, respectively, which were in agreement with the percentage of explanation due to sow vaccination (R^2^ = 41.2% for the weighted and R^2^ = 11.5% for the unweighted distance). In all these analyses, sow vaccination explained a higher percentage of variation when the analysis included quantitative data.

### Taxonomical assignment and differential abundance analysis

**Figure 3:**
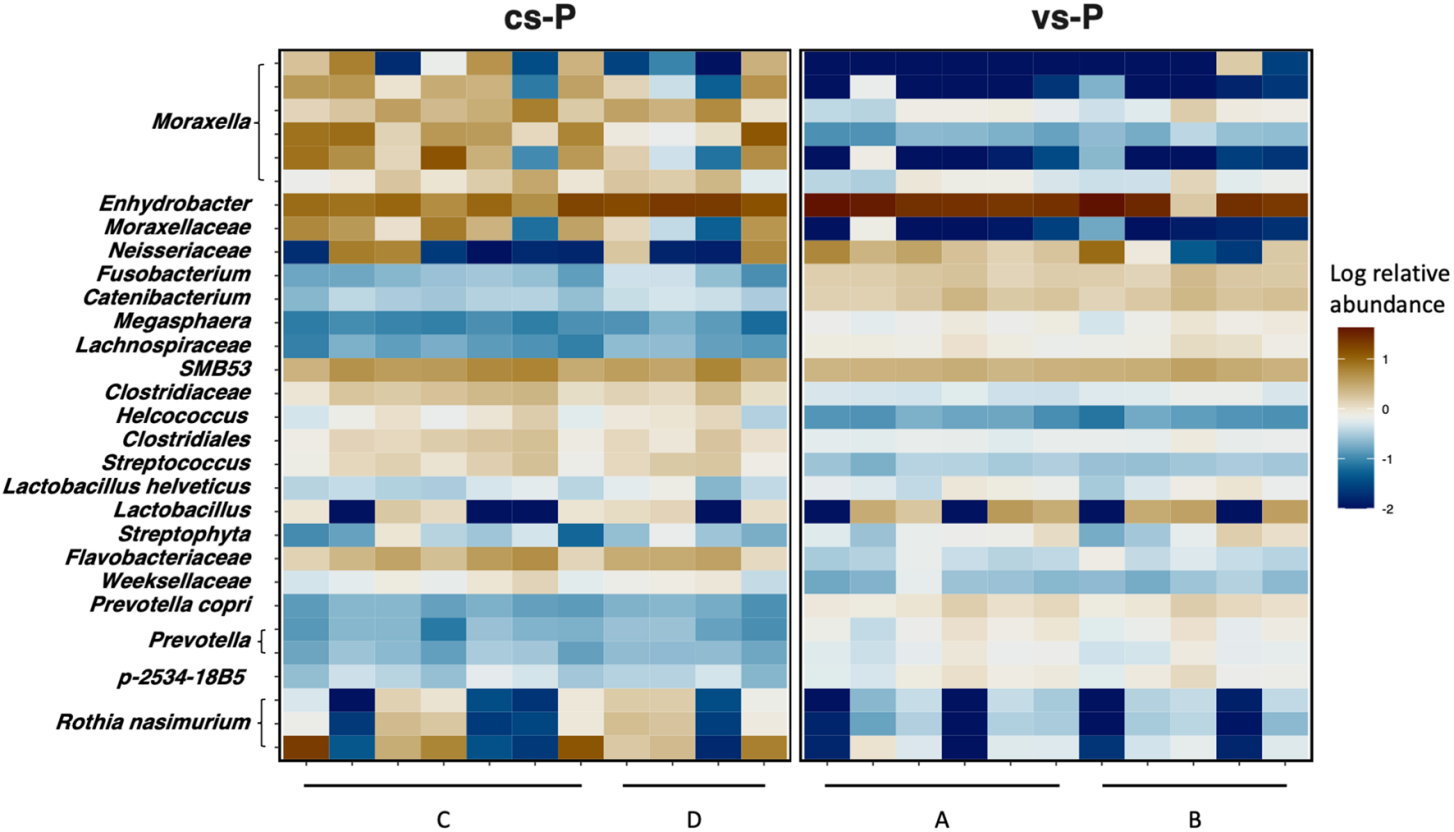
Heatmap representing the top 30 most relative abundant ASVs globally. The relative abundance (log10) of each ASV in the nasal microbiota of piglets from control sows (cs-P) and vaccinated sows (vs-P) is shown. The biological mothers are indicated in the bottom as A, B, C and D.

The denoised sequences corresponded to 9817 ASVs with different taxonomic assignment. Taxa assignation at the different levels showed a percentage of non-assigned taxa: 3.1% at phylum and 5.05% at genus. At phylum level, *Firmicutes*, *Proteobacteria* and *Actinobacteria* were the most relative abundant taxa in both cs-P and vs-P groups, while the ten most relatively abundant genera were *Enhydrobacter*, *SMB53*, *Rothia*, *Moraxella*, *Neisserieceae sp*., *Lactobacillus, Clostridaceae sp*., *Catenibacterium*, *Prevotella* and *Fusobacterium*. The top 30 most relatively abundant ASVs among all the samples from both groups were heterogenously distributed within each group and included 6 ASV classified as *Moraxella*, 3 as *Rothia*, 2 as *Prevotella*, and the rest of taxa represented by a unique ASV (Fig. 3).

To unravel the differences in bacterial communities inhabiting the nasal microbiota from each group, we used two different approaches. First, the analysis of composition of microbiomes (ANCOM) showed 21 ASVs that differed between each study group (Supplementary Table S1). Eighteen of those ASVs were more abundant in the control group, and included *Moraxella*, *Staphylococcus*, *Prevotella* and *Actinobacillus*; all with relative abundances lower than 0.33%. ANCOM was also performed at different taxonomic levels and detected ten different phyla that were differentially abundant according to the study group. Seven out of these ten were increased in the vs-P group: *Tenericutes*, *SR1*, *Planctomycetes*, *Euryarcheota*, *Lentisphareae*, *TM7*, *Fibrobacteres*, *Fusobacteres*, *Verrucomicrobia*, *Spirochaetes*, and *Actinobacteria*. On the other hand, the phyla that were more abundant in the cs-P group were *GN02*, *Firmicutes*, and *Bacteroidetes*. At genus level, *Selenomonas*, *Megasphaera*, *Akkermansia*, and *Anaeroplasma* were associated with the vs-P group.

**Figure 4:**
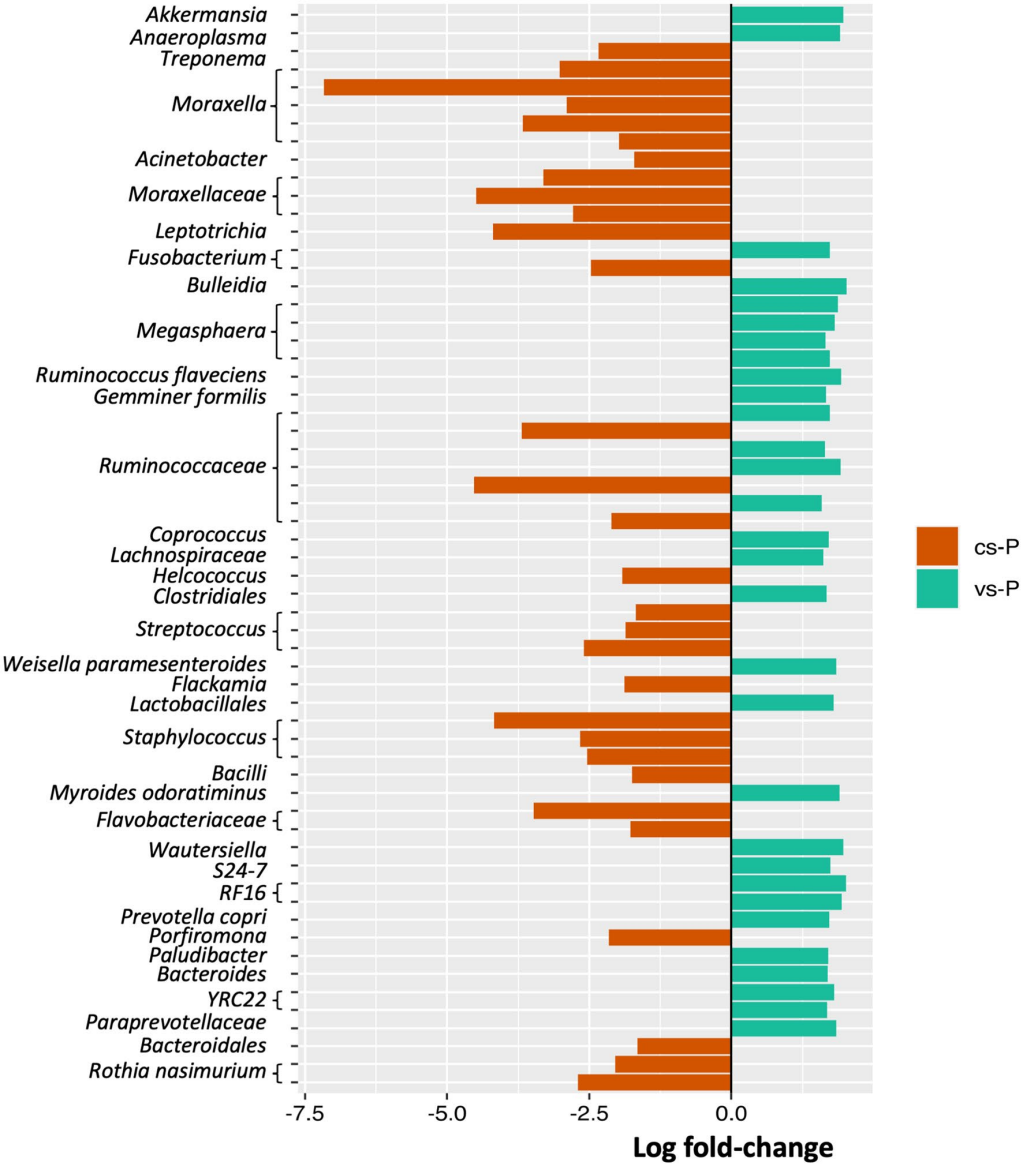
Differential abundant features between nasal microbiota composition of piglets from control (cs-P, orange bars) or vaccinated sows (vs-P, green bars). The association of the 30 features with the highest differential ranked coefficient estimated with *q2-songbird* is depicted (log fold-change).

Second, the differential ranking *Songbird* method was used as a complementary method to infer taxa associated to either cs-P or vs-P group, showing a list of 1509 ASVs. Once the low relatively abundant ASVs (less than 0.1% in either group) were removed, 186 ASVs showed a statistically significant differential abundance, with 83 of them associated to the cs-P group and 103 ASVs associated to the vs-P group. The differential abundant ASVs included the 30 most abundant ASVs across all the groups, whose association to either vs-P or cs-P is depicted in Supplementary Figure S2. The 30 ASVs most associated to the vs-P group included ASVs classified as *Ruminococcaceae* (4 ASVs), *Megasphaera* (4 ASVs), *Paraprevotellaceae* (3 ASVs) and *Lachnospiraceae* (2 ASVs). In contrast, among the 30 most associated to the cs-P group, we found ASVs assigned to *Moraxella* (5 ASVs), *Moraxellaceae* (3 ASVs), *Ruminococcaceae* (3 ASVs), *Streptococcus* (3 ASVs), *Staphylococcus* genus (1 ASV), *Flavobacteriaceae* (2 ASVs) and *Rothia nasimurium* (2 ASVs) (Fig. 4). In general, the differential ASVs classified within a given taxon seemed to be predominantly associated to one group or the other, e.g., *Moraxella* ASVs were associated with cs-P, while *Megasphaera* ASVs were associated with vs-P (Fig. 4). Among the most relatively abundant ASVs (Supplementary Figure S2), twelve were shared with the most associated to each group (with high log-fold change value) corresponding to the following taxa: *Rothia nassimurium* (2 ASVs), *Prevotella copri*, *Flavobacteriaceae*, *Helcococcus*, *Lachnospiraceae*, *Megasphaera*, *Fusobacterium*, *Moraxellaceae* and *Moraxella* (3 ASVs).

Since the vaccine used targeted virulent strains of *G. parasuis*, we focused our analysis on the potential effect of this vaccine on this species. We found a total of ten different ASVs classified as *G. parasuis,* which in global showed lower relative abundance in the vs-P group than in the cs-P group (*G. parasuis* relative abundance: 0.058% ± 0.029 for vs-P and 0.225% ± 0.112 for cs-P). From these 10 *G. parasuis* ASVs, 5 were statistically associated to the control group, where three ASVs were exclusively found in the cs-P group. These findings were found by the Songbird differential rank analysis, but they were not detected using ANCOM.

### Correlation network analysis

Correlation Network Analysis (CNA) at ASV level, resulted in two different undirected graphs when samples from the different groups were analyzed separately. The topology of the two graphs showed different characteristics. The cs-P network had 3,552 nodes with 1,678,545 edges and a network density of 0.266, while the vs-P network had 3,048 nodes with 1,324,390 edges and a network density of 0.285. On the other hand, the diameter of both networks was three.

The top ten most-connected nodes in the cs-P network ranged between 1,640 and 1,670 edges, and they were grouped in three different modules. Module 6 included ASVs from *Actinobacillus*, *Ruminococcus flavefaciens, Erysipelotrichaceae, Staphylococcus*, *Glaesserella parasuis* and *Filifactor* out of 76 total component nodes; module 8 (67 ASVs) included *WCHB1-25* and *Corynebacterium,* while module 13 (37 ASVs) included *Oscillospira*, and *Ruminococcus gnavus*. On the other hand, the most connected nodes in the vs-P were grouped in five different modules. Module 1 (124 ASVs) included the following most-connected taxa: *Lachnospiraceae* and *Clostridiales*; whereas *Coprococcus*, *Bacteroidales* and *Sphaerochaeta* taxa were highly-connected members of module 2 (91 ASVs). ASVs from *Moraxellaceae*, *Neisseriaceae* and *Moraxella* belonged to module 12 (37 ASVs), while the last two modules included *Oscillospira* in module 32 (23 ASVs) and *Lachnospiraceae* in module 41 (20 ASVs). None of the 10 most-connected nodes in the two networks was shared.

**Figure 5:**
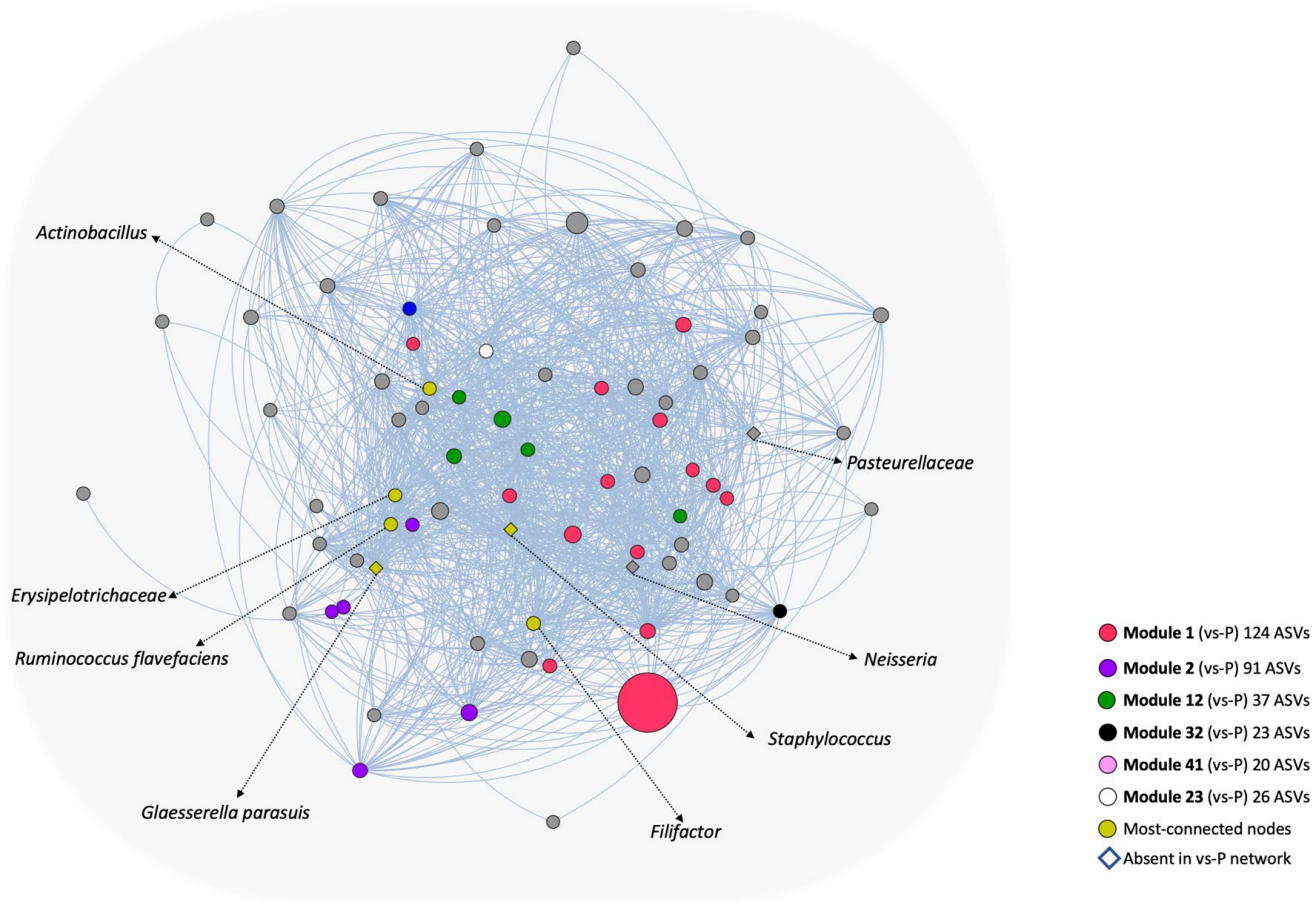
Subnetwork representing the module 6 of the co-occurrence network from the nasal microbiota of piglets from control sows (cs-P). Each node corresponds to ASVs and edges in the network represent the positive correlations between the different nodes. The nodes are colored based on the location of these ASVs in the network of the nasal microbiota composition of piglets from vaccinated sows (vs-P) modules. ASVs, in this module 6 from the cs-P network, that are present as the top ten most-connected nodes in the vs-P network, are represented in different colors regarding the modules they belong in the vs-P network: red, nodes in vs-P module 1; green, nodes in vs-P module 12; purple, nodes in vs-P module 2; black, node in vs-P module 32, and pink, for node in module 4. Yellow color highlights the most connected nodes in this module 6 from the cs-P network (6 nodes). The white node corresponds to an ASV classified as *Erysipelotrichaceae,* which is located in the same module as a *G. parasuis* ASV in the vs-P network. Diamond shaped nodes correspond to the four nodes that were absent in the vs-P network. Size of the nodes are proportional to their relative abundance, ranging from 0.0039% (*Neisseria*, one of the diamond shaped nodes) and 5.07% (*SMB53*, largest red-colored node).

Among all the modules containing the ten most-connected nodes, module 6 from cs-P network had the highest number of these highly connected nodes, including an ASV classified as *G. parasuis* (Fig. 5). When we compared the networks, we found that the ASVs from module 6 in the cs-P network, clustered differently in the vs-P network. From the 76 ASVs composing the module 6 from the cs-P network, 72 were found in 31 different modules in the vs-P network. Noteworthy, among these 31 modules, we found the module 23 containing an ASV classified as *G. parasuis,* reinforcing that the rearrangements in the community structure were due to vaccination against this pathogen. Five out of these 31 modules contained the top-ten most-connected nodes in the vs-P network (Fig. 5). The remaining 4 nodes, assigned to *G. parasuis*, *Neisseria*, *Staphylococcus* and *Actinobacillus porcitonsillarum*, were absent in vs-P network.

### Metagenome prediction and functional analysis inference

**Figure 6:**
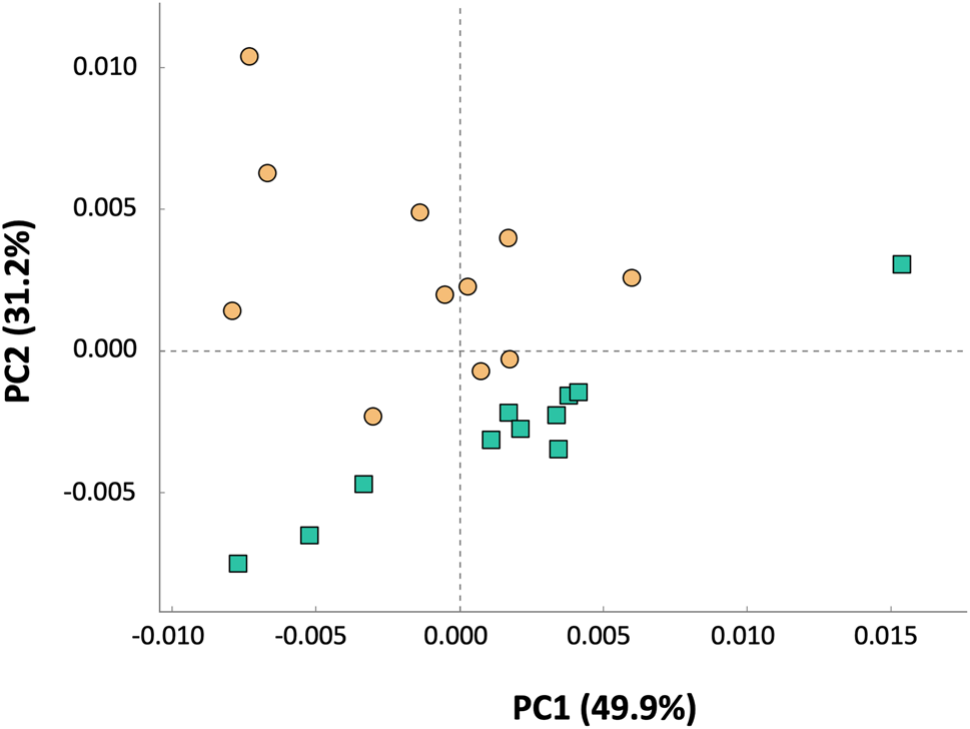
PCA of the functional pathway composition of each sample. Samples from piglets from vaccinated sows (vs-P group) are represented as green squares and samples from piglets from control sows (cs-P group) are represented as oranges circles.

**Figure 7:**
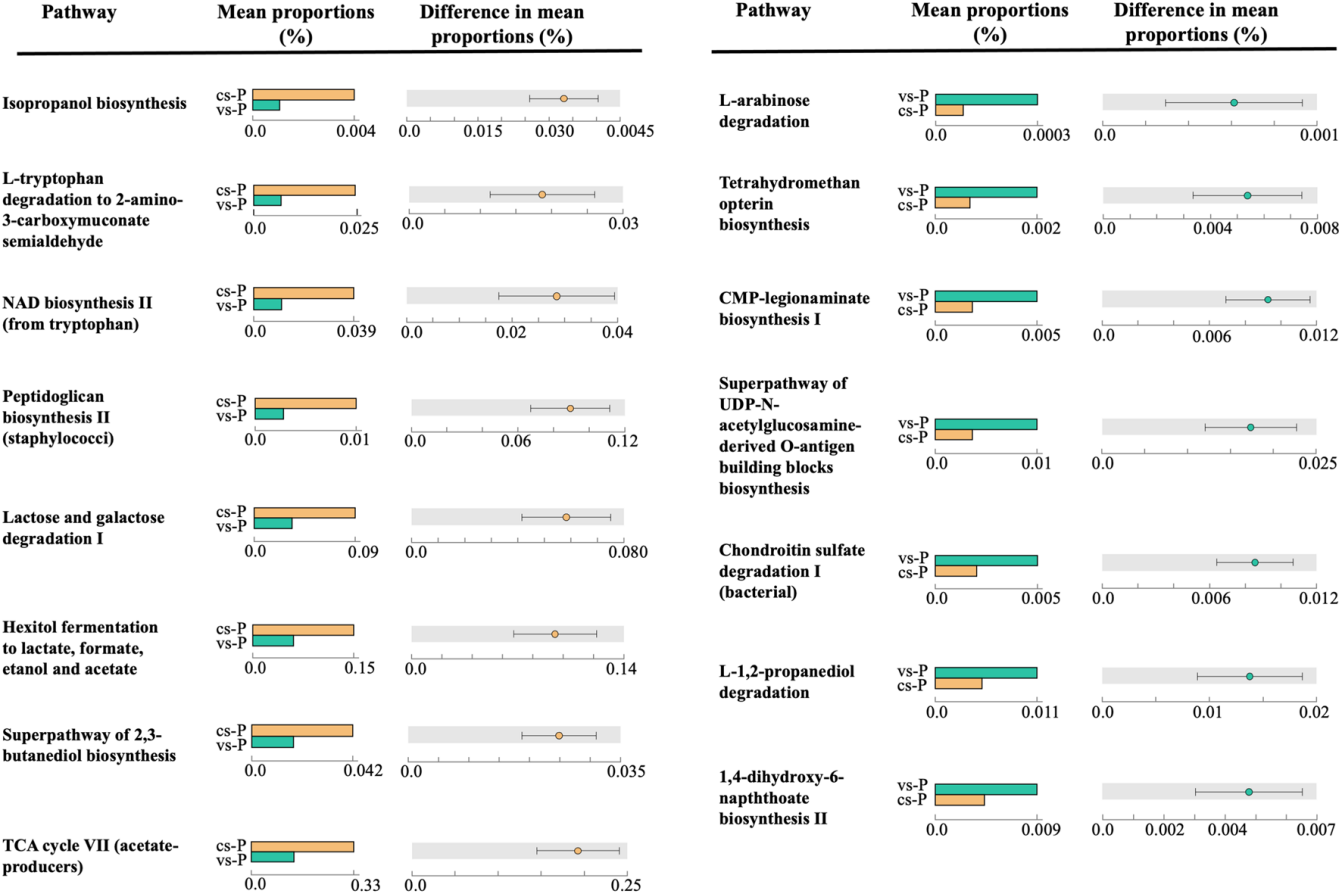
Predicted KEGG functional pathways using PICRUSt analysis. Most statistically significant differential pathways between the nasal microbiota of piglets from vaccinated (vs-P) and control sows (cs-P) are shown (Kruskal–Wallis *post-hoc* test with Bonferroni correction). Pathways more relatively abundant in the cs-P samples are shown on the left, and those more relatively abundant in the vs-P samples are shown on the right.

The metagenome was predicted from the 16S partial sequences in the two study groups to infer the potential functionality of the microbiota using the KEGG public database. PCA of the functional pathways showed a robust clustering of the samples by the group they belonged (Fig. 6), in agreement with the composition (beta diversity) analysis (Fig. 2) and suggesting that the microbial composition changes led to functional changes. Only 32 functional pathways were significantly different between the two study groups (Kruskall Wallis test, *P* value with Bonferroni correction < 0.05, Supplementary Table S2), where 15 of them presented a two-fold change (Fig. 7). The most relatively abundant pathways in the cs-P group were the isopropanol biosynthesis and L-tryptophan degradation to 2-amino-3-carboxymuconate semialdehyde, while the most relatively abundant in the vs-P group were L-arabinose degradation IV and the tetrahydromethanopterin biosynthesis. Pathways related with the biosynthesis with the peptidoglycan were in higher abundance in the cs-P group and with the biosynthesis of the LPS was higher in the vs-P group.

## Discussion

Vaccination against specific pathogens has been an extensive method to control infectious diseases for a long time now. Primary function of all vaccines is the stimulation of the immune system against specific antigens, while one of the main tasks of the microbiota is to contribute to the maturation of the immune system^17–20^. Thus, the influence of the microbiota on the vaccine response has been more studied than the effect that the vaccines may have in the host microbiota and, in particular, in the microbiota of the progeny^16,21^. Here, we have shown that sow vaccination modified the nasal microbiota composition of their piglets with an effect extended beyond the targeted pathogen.

Vaccination reduces the shedding of the targeted pathogens^14^. Accordingly, here we observed a reduction of the relative abundance of all the *G. parasuis* ASVs after vaccination. Some of these ASVs classified as *G. parasuis* were statistically associated with the control group, while two of them were absent in piglets born to vaccinated sows. This reduction may be due not only to the decreased shedding from the mother but also by the maternal immunity transferred through the colostrum to the piglets as specific antibodies^22^. As the vaccine used was designed against virulent strains of *G. parasuis*, it would be very interesting to determine if in fact, the virulent strains were specifically reduced in the piglets from vaccinated sows. Additional analyses, including shotgun sequencing metagenomics, may provide better resolution to study the different strains (variants) in the microbiota.

Sow vaccination also affected the general composition of the piglets’ nasal microbiota. The nasal microbiota of the offspring from vaccinated sows showed a slight decrease in alpha-diversity. This was especially observed with the Simpson index (biased towards abundant features^23,24^), indicating that the bacterial communities that are in relatively higher abundance in the nasal cavity of piglets drove the differences observed in our groups. In agreement, beta diversity analysis showed a higher percentage of explanation of the clustering in the indexes that include the quantity of the ASVs. Taken together, our findings indicate that vaccination of the mother did not change the main components of the nasal microbiota in the offspring but caused a clear disparity in the distribution of the taxa. In the differential abundance analysis, we observed that Songbird, a differential rank approach, discovered many ASVs that were not detected by ANCOM (1509 ASVs detected by Songbird *vs* 21 ASVs by ANCOM). It was reported that ANCOM fails to infer differential microbes in some composition scenarios, where these are actually changing^25^. The top ranked ASVs (most positive or negatively correlated to sow vaccination) within a given taxa tended to be associated to only one of the groups. Thus, ASVs from *Moraxella*, *Streptococcus*, *Staphylococcus* and *Rothia* were associated with cs-P, while ASVs classified as *Megasphaera*, *YRF22* and *RF16* were associated with the vs-P group. However, this specific association of the ASVs from one taxon to one group was not observed in other ASVs, reinforcing the importance of detailed analyses at the ASV level. Strains within a given taxon can be heterogeneous, as demonstrated for *Glaesserella*^26,27^ or *Moraxella* spp. nasal isolates^28^, and therefore they can play a different role in the microbiota.

Correlation Network Analysis has been a useful tool to identify global relationships between the microorganisms that compose the microbiota. Here, we observed that the network of piglets from vaccinated sows had lower complexity, demonstrated by the lower number of nodes, modules and edges, when compared to the control network. The structure of the modules containing the most connected nodes from the control network were not conserved in the structure shaped by the vaccination of the sows. Both the lower shedding of the pathogen by the mother, which in turn may produce a reduction of the pathogen in the piglet, and the effect of the maternal immunity transferred by the colostrum, might contribute to this rearrangement of the network topology.

The strong clustering between the two groups of piglets observed in the beta diversity analyses, resulted in a clear differential predicted metagenome, i.e., differential predicted functionality. Some of the metabolic functional pathways that were in higher abundance in the control group, such as isopropanol and L-tryptophan degradation are related to an immune suppressive status in mammals, as reported in the literature. On the other hand, none of the pathways in the vs-P group was related to any function of the immune system. The changes that we observed in the functionality may affect the health status or the immune response in the future, but this is difficult to assess and deserves further exploration. In general, the ASVs assigned to taxa of potential pathogens (*Staphylococcus*, *Neisseria*, *Streptococcus* and *Mycoplasmataceae*) were negatively correlated with the vs-P group, suggesting that the reduction of virulent strains of *G. parasuis* may contributed to drag out other associated pathogens in the nasal cavity^26,29,30^. Moreover, potentially beneficial bacteria, such as *Prevotella*, were associated with the vs-P group. Members of the *Prevotella* genus have been associated to positive outcomes in pig production, including growth performance and immune response^31,32^. Indeed, 122 from the 172 ASVs classified as *Prevotella* were associated with sow vaccination.

In summary, sow vaccination with a peptide directed against virulent *G. parasuis* influenced the nasal microbiota of the offspring, not only reducing the relative abundance of the targeted pathogen but also modifying the balances in general composition of the microbiota during at least 15 days of life. These changes in nasal microbiota composition are expected to affect its functionality.

## Materials and methods

### Study design and sampling

The experimental study was described previously by López-Serrano et al.^5^ and was performed under the approval of the Ethics Commission in Animal Experimentation of the Generalitat de Catalunya (Protocol number 9211) and was carried out in compliance with the ARRIVE guidelines. Briefly, four pregnant sows were included in the study based on low antibody levels against *G. parasuis*. Two sows were vaccinated intramuscularly with 2.5 ml of 100 μg/mL F4 (an antigen that is specific of virulent strains of *G. parasuis*) in combination with Carbopol 5984 EP polymer adjuvant (Lubrizol, Cleveland, OH, USA), while the other two sows remained unvaccinated and were used as control. Vaccination was performed at 32 and 12 days pre-farrowing. Newborn piglets were kept with their biological mother during their first hours of life (at least 12 h). Twenty-four hours after delivery, only one sow from each group was maintained in the study, with biological piglets and cross-fostered piglets from the other sow from the same study group. Nasal swabs were taken from 11 piglets from vaccinated sows (vs-P) and 11 piglets from control sows (cs-P) at 15 days of life. Nasal swabs were resuspended in 500 μl de PBS, vortexed for 30 s and stored at -80ºC until used for microbiota analysis.

### DNA extraction and 16S rRNA gene amplicon sequencing

DNA was extracted from 200 μl of resuspended swabs using the Nucleospin Blood (Macherey–Nagel) kit. Quantity and quality assessment of the DNA was performed using BioDrop DUO (BioDrop Ltd). DNA samples were submitted to the Servei de Genòmica (Universitat Autònoma de Barcelona) for 16S rRNA amplicon sequencing using Illumina MiSeq pair-end 2 × 250 bp technology following the manufacturer instructions (MS-102–2003 MiSeq Reagent Kit v2, 500 cycle). The region amplified covered the V3-V4 hypervariable regions^33^. The complete dataset from this study is available at the NCBI database, under SRA accession number PRJNA783272.

### Microbiota analysis

Bioinformatics analysis was done using the Quantitative insights into microbial ecology (Qiime2) platform^34^. Quality control step was done using *q2-dada2* plugin, where all the reads with a Phred quality-value under 20 and a length under of 234 bp in the forward read and 229 bp in the reverse read, were removed. Chimeric-reads check was also done and were removed for further analysis. In addition to the quality-control step, an alignment against the reference Greengenes (version 13.8) 16S rRNA gene database^35^ was done to remove sequences not matching with an identity of 80% and a query length of 50%. Richness and alpha diversity analysis were done using Observed Features (in this case, Amplicon Sequence Variants, ASVs)^36^, Shannon index^36^, Chao index^37^, Simpson Index and Simpson evenness^38^. Different beta diversity metrics were used to assess the diversity across the samples, both qualitatively with Jaccard similarity coefficient^39^ and quantitatively using Bray Curtis dissimilarity index^40^. Moreover, phylogenetic metrics weighted and unweighted Unifrac^41^ were calculated as well. These distance matrixes were used to perform Principal Coordinate Analysis (PCoA) using *core-metrics* plugin and a PERMANOVA test was done to analyze the clustering of the sample groups. To extract the percentage of variations explained by each metadata column, the Adonis function was performed on every distance matrix using *Vegan*^42^ package. Taxonomic assignation of each amplicon sequence variant was done using the Qiime2 classifier trained with the V3-V4 region from 16S gene and the Greengenes database (13.8 version)^35^. Differential abundant analysis was done using the analysis of composition of microbiomes (ANCOM)^43^ and the Songbird differential ranking^25^ algorithms.

Metagenome prediction from the 16S rRNA sequencing was done using PICRUSt2^44^. This software aligns the ASVs and place them in a tree (EPA-NG, GAPPA) with reference sequences and it infers the family genes and function pathways from KEGG^45^ orthologs and Enzyme Classification numbers. Together with the gene family copy number references, it predicts the gene content per ASV and with the abundance of each ASV in the study dataset it can determine the gene family abundance per sample. Mapping the gene family to pathways abundances was done using MinPath^46^ integrated in the PICRUSt2 pipeline. Statistical Analysis of Microbial Profiles (STAMP)^47^ software was used to visualize and perform statistical analysis over the predicted functional pathways outputted by PICRUSt2. Statistical analysis was based on a Kruskal Wallis test and Tukey–Kramer *post-hoc* test of the functional pathways of the different study groups were done using a Kruskal Wallis^48^ test and a Tukey–Kramer *post-hoc* test^49^. After a correction with Bonferroni *P* values under 0.05 were considered significant.

We built two separate networks from the ASVs of each group samples (cs-P and vs-P) Correlation Network Analysis (CNA) was performed using the Sparse Co-occurrence Network Investigation for Compositional data (SCNIC) integrated in the *q2-scnic* plugin from Qiime2^50^ software toolkit. All the samples that had less than 500 features and also all the features that had an average mean less than 2 across all the samples, were filtered out. Pairwise correlations between all our ASVs in our dataset were calculated with *sparCC*^51^ algorithm. From that correlation table, we built our network with a minimum of R correlation-value cutoff of 0.35. Detection of modules was done by the summarization and clustering high connected nodes. Output files with nodes membership and edged connections were analyzed in Cytoscape^52^ software and extracted to tables to further data analysis to query ASVs and nodes. These tables were processed in Rstudio^53^ and merged with previous results using *tidyverse* package^54^.

All the plots and figures were done using R script language. Rstudio^53^ was used as a coding work environment. The packages used to build the plots and figures were specifically: *qiime2R*^55^*, ggplot2*^56^ and *tidyverse*^54^.

### Ethics statement

The animal study was performed in the BSL3 animal facilities of IRTA-CReSA (Bellaterra, Spain) following proper veterinary practices, in accordance with European (Directive 2010/63/EU) and Spanish (Real Decreto 53/2013) regulation and with the approval of the Ethics Commission in Animal Experimentation of the Generalitat de Catalunya (Protocol number 9211).

## Supplementary Information

Supplementary Information 1.

Supplementary Information 2.

Supplementary Information 3.

## Data Availability

The complete dataset from this study is available at the NCBI database, under SRA accession number PRJNA783272 available at https://www.ncbi.nlm.nih.gov/sra/PRJNA783272.
