# Supplementary material for: Sow vaccination against virulent Glaesserella parasuis shapes the nasal microbiota of their offspring
Source: Sci Rep. 2022 Mar 1;12:3357. doi: 10.1038/s41598-022-07382-2 (PMC8888576; doi:10.1038/s41598-022-07382-2)
Supplement: Supplementary file 1 — Supplementary Information 1. [file 41598_2022_7382_MOESM1_ESM.pdf]

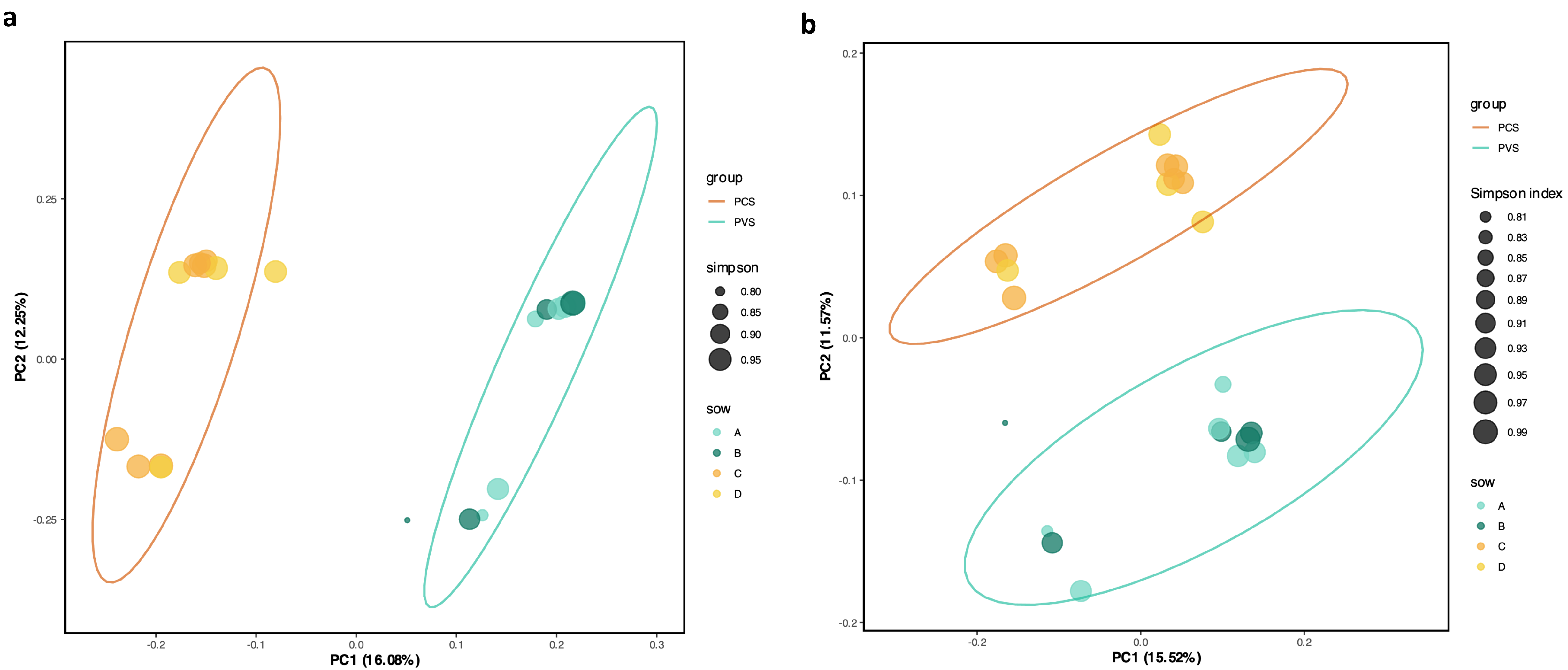

Figure S1. PCoA plots representing the beta diversity of the nasal microbiota of the piglets derived from vaccinated (green) and control sows (orange). Jaccard distance matrix is represented in the left (a) and unweighted Unifrac index on the right (b). Each sample is colored based on the biological sow, and its size is proportional to the Simpson index (alpha diversity).

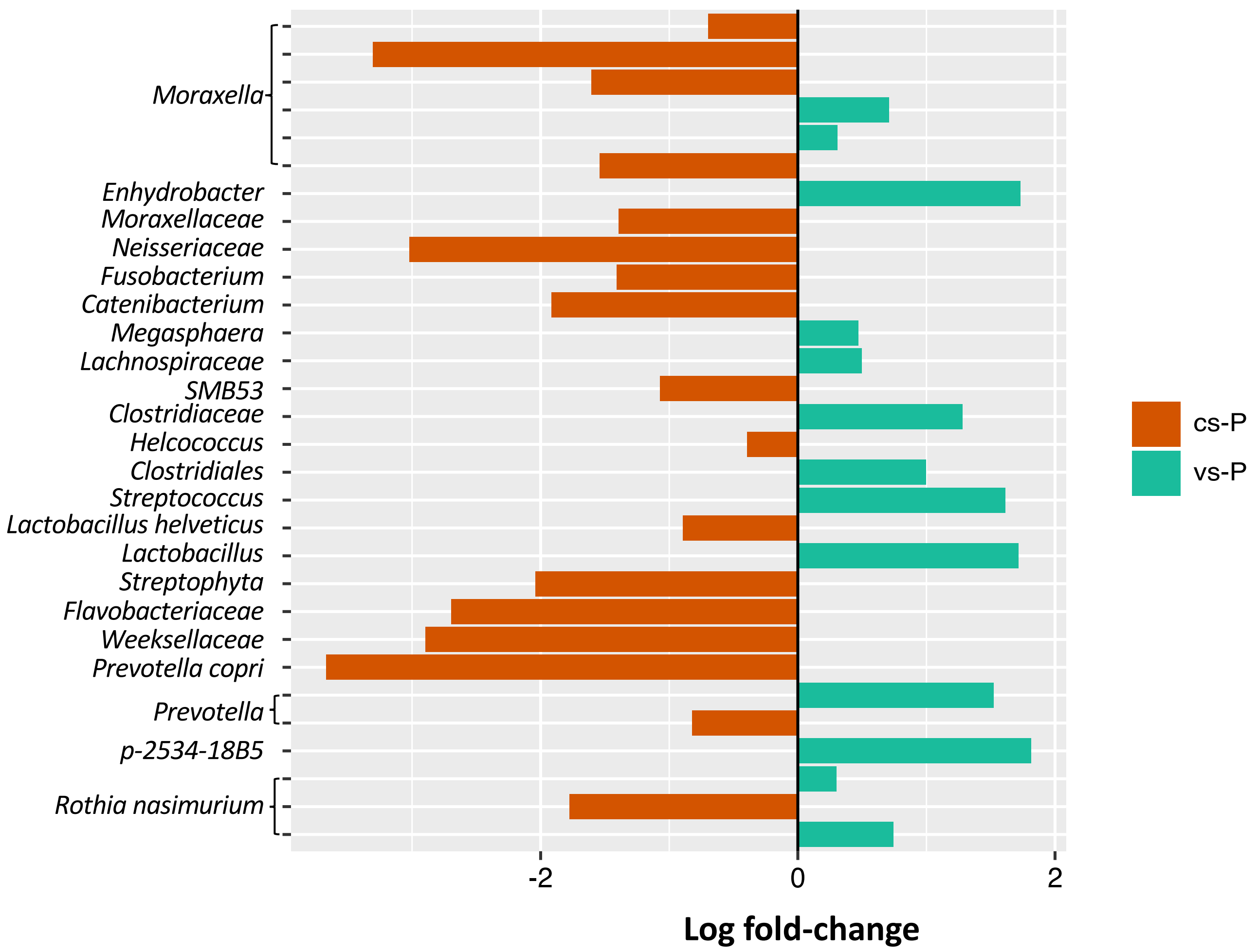

Figure S2. Differential abundant features between nasal microbiota composition of piglets from control (cs-P, orange bars) or vaccinated sows (vs-P, green bars). The association of the top 30 most relative abundant ASVs across all the samples estimated with *q2-songbird* is depicted (log fold-change).
